# CT radiomics to differentiate between Wilms tumor and clear cell sarcoma of the kidney in children

**DOI:** 10.1186/s12880-023-01184-2

**Published:** 2024-01-05

**Authors:** Yaxin Deng, Haoru Wang, Ling He

**Affiliations:** Department of Radiology, Children’s Hospital of Chongqing Medical University, National Clinical Research Center for Child Health and Disorders, Ministry of Education Key Laboratory of Child Development and Disorders, Chongqing Key Laboratory of Pediatrics, Chongqing, 400014 China

**Keywords:** Children, Clear cell sarcoma of the kidney, Computed tomography, Radiomics, Wilms tumor

## Abstract

**Background:**

To investigate the role of CT radiomics in distinguishing Wilms tumor (WT) from clear cell sarcoma of the kidney (CCSK) in pediatric patients.

**Methods:**

We retrospectively enrolled 83 cases of WT and 33 cases of CCSK. These cases were randomly stratified into a training set (*n* = 81) and a test set (*n* = 35). Several imaging features from the nephrographic phase were analyzed, including the maximum tumor diameter, the ratio of the maximum CT value of the tumor solid portion to the mean CT value of the contralateral renal vein (CTmax/CT renal vein), and the presence of dilated peritumoral cysts. Radiomics features from corticomedullary phase were extracted, selected, and subsequently integrated into a logistic regression model. We evaluated the model's performance using the area under the curve (AUC), 95% confidence interval (CI), and accuracy.

**Results:**

In the training set, there were statistically significant differences in the maximum tumor diameter (*P* = 0.021) and the presence of dilated peritumoral cysts (*P* = 0.005) between WT and CCSK, whereas in the test set, no statistically significant differences were observed (*P* > 0.05). The radiomics model, constructed using four radiomics features, demonstrated strong performance in the training set with an AUC of 0.889 (95% CI: 0.811–0.967) and an accuracy of 0.864. Upon evaluation using fivefold cross-validation in the training set, the AUC remained high at 0.863 (95% CI: 0.774–0.952), with an accuracy of 0.852. In the test set, the radiomics model achieved an AUC of 0.792 (95% CI: 0.616–0.968) and an accuracy of 0.857.

**Conclusion:**

CT radiomics proves to be diagnostically valuable for distinguishing between WT and CCSK in pediatric cases.

**Supplementary Information:**

The online version contains supplementary material available at 10.1186/s12880-023-01184-2.

## Background

Clear cell sarcoma of the kidney (CCSK) is a rare renal malignancy, accounting for approximately 2–5% of pediatric renal tumors, ranking second in prevalence after Wilms tumor (WT). Typically, CCSK manifests between the ages of 2 and 4 years [1, 2]. Despite its low incidence, CCSK frequently leads to bone metastases, resulting in a prognosis less favorable than that of WT [3, 4]. Late recurrence often occurs in the brain for CCSK cases. However, recent advancements in chemotherapy and radiotherapy have significantly improved CCSK outcomes. Clinically and radiologically, distinguishing CCSK from WT can be challenging, with differential diagnosis often relying on histopathological analysis and immunophenotyping [5–7]. However, biopsies are limited in their ability to capture a small fraction of tumor heterogeneity [8]. The diverse histology of CCSK, coupled with a lack of reliable immunohistological markers and limited effectiveness of molecular genetics, often results in pathological examinations that do not fully elucidate the tumor biological characteristics [4, 9].

Radiomics presents a promising avenue for tumor detection and differential diagnosis by unveiling concealed information that extends beyond the capabilities of the human eye. It accomplishes this by quantifying pixel distribution within medical images, thereby capturing the comprehensive heterogeneity of the entire tumor [10]. Prior studies have revealed the valuable utility of CT radiomics in pediatric extracranial solid tumors such as neuroblastoma and rhabdomyosarcoma [11–14]. While radiomics analysis has been extensively applied in the context of adult renal tumors in previous literature [15, 16], the use of texture analysis based on medical imaging to identify pediatric renal malignancies remains limited, with only one previous study investigating ultrasound-based texture analysis in this domain [5]. Recent study has demonstrated that specific qualitative and semi-quantitative imaging features derived from contrast-enhanced CT can effectively differentiate between cases of CCSK and WT [7]. Consequently, the application of radiomics analysis to contrast-enhanced CT images holds substantial promise for providing additional imaging biomarkers that facilitate the differentiation of CCSK from WT.

Hence, the primary objective of this study was to differentiate between CCSK and WT in pediatric patients utilizing radiomics features derived from contrast-enhanced CT images.

## Methods and Materials

### Patients

**Fig. 1:**
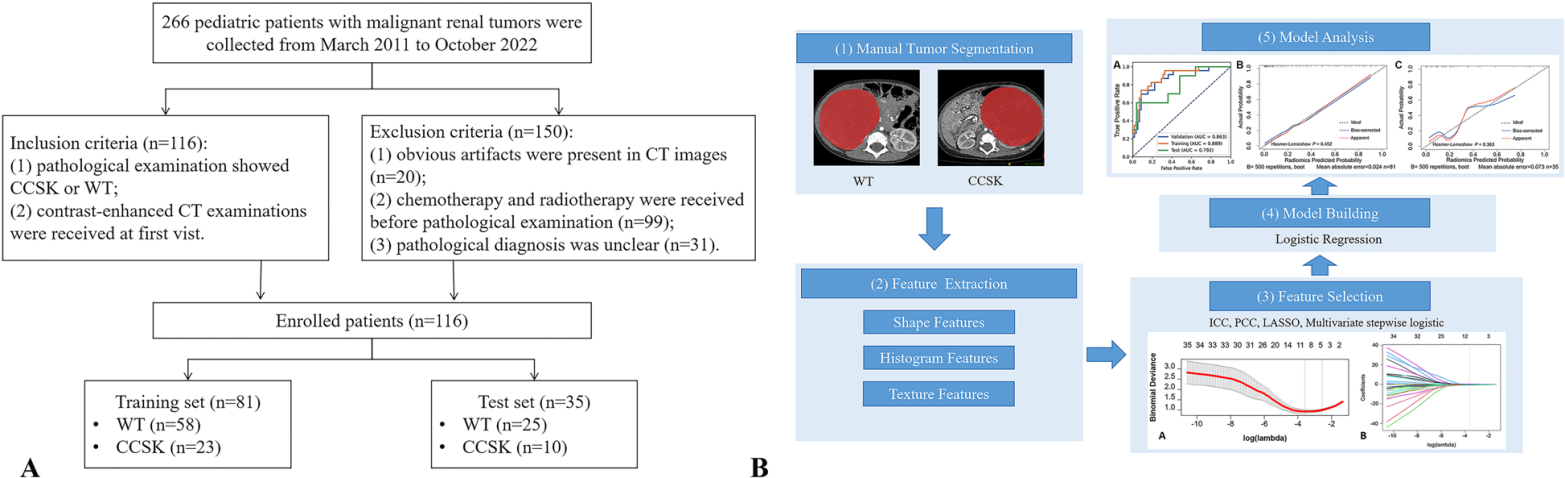
An overview of the system diagram of patient selection (Figure **A**) and radiomics workflow (Figure **B**) in this study

This retrospective study received approval from the Institutional Review Board of Children's Hospital of Chongqing Medical University, and patient informed consent requirements were waived. All methods were performed in accordance with the relevant guidelines and regulations or declaration of Helsinki. Patients diagnosed with WT and CCSK between March 2011 and October 2022 were retrospectively enrolled (Fig. 1A). Inclusion criteria included: (1) Confirmation of CCSK or WT through pathological examination; (2) Undergoing preoperative contrast-enhanced CT examinations. Exclusion criteria were as follows: (1) The presence of noticeable artifacts in CT images; (2) Prior utilization of chemotherapy and radiotherapy before pathological diagnosis; (3) Cases with unclear pathological diagnoses. Among them, 116 patients—comprising 83 cases of WT and 33 cases of CCSK—were randomly stratified into training and test sets using a 7:3 ratio using the open-source software FeAture Explorer (version 0.5.5) [17]. Therefore, there were 58 cases of WT and 23 cases of CCSK in the training set, and there were 25 cases of WT and 10 cases of CCSK in the test set.

### Examination method

Patients participating in this study were evaluated under calm conditions. In cases where patients were unable to cooperate with the CT examination, 10% chloral hydrate (0.5 mL/kg of body weight) was administered orally, or sodium phenobarbital (5 mg/kg of body weight) was administered intramuscularly. The scanning parameters used were as follows: slice thickness of 5.0 mm, slice spacing of 5.0 mm, automatic voltage, and automatic tube current. A 320 mg I/mL iodine contrast agent was chosen and administered at a dose of 1.0–2.0 mL/kg. Subsequently, the contrast agent was injected intravenously, and the corticomedullary and nephrographic phase scans were conducted 15–30 s and 55–65 s after the injection of the contrast agent, respectively.

### Region of interest segmentation

**Fig. 2:**
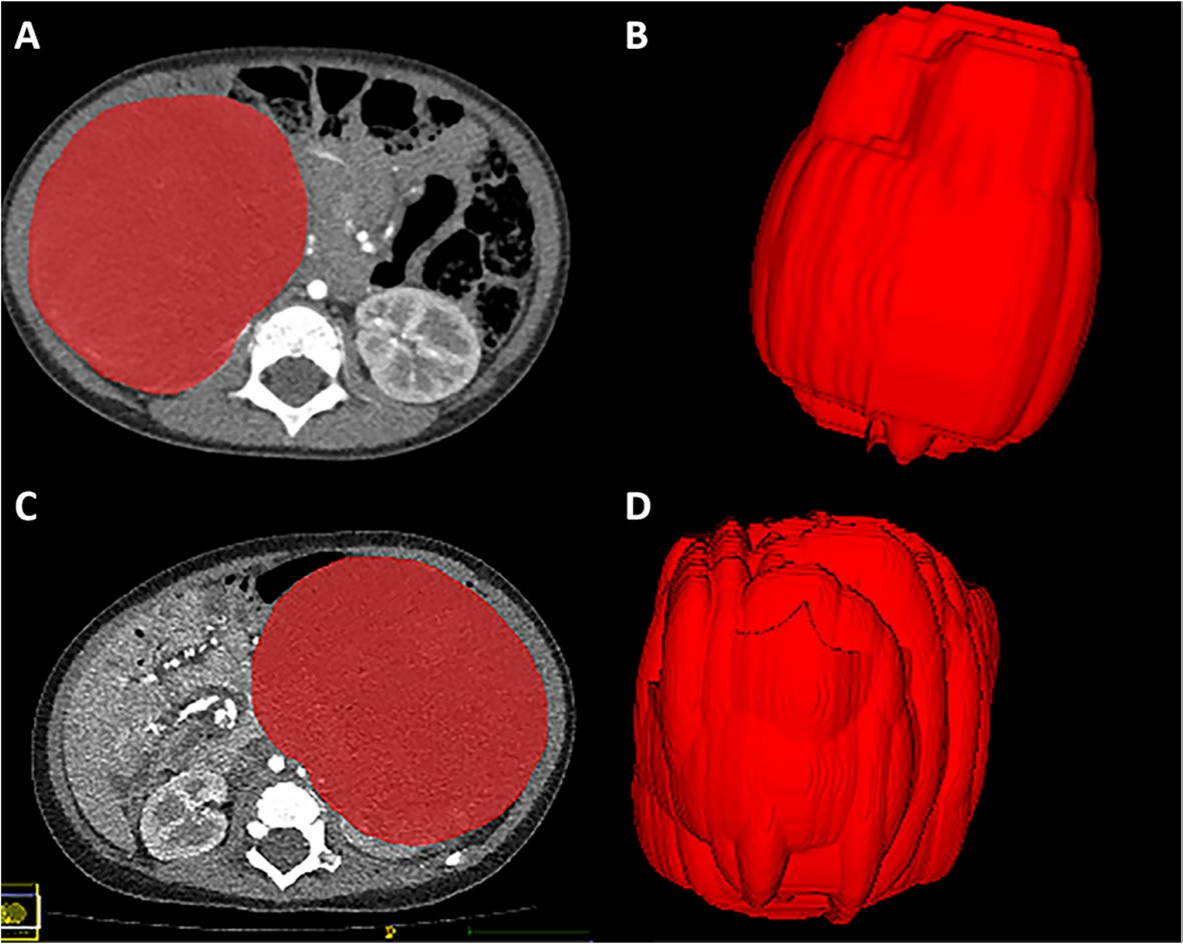
Examples of tumor region of interest outlining

The radiomics workflow is demonstrated in Fig. 1B. The corticomedullary phase CT images were retrieved from the Picture Archiving and Communication System (PACS), and image segmentation was conducted using an open-source ITK-SNAP software (version 3.6.0). A radiologist, blinded to the patient's clinical details, manually delineated the tumor boundaries. Subsequently, a senior radiologist reviewed and verified these areas (Fig. 2). To assess the agreement and reproducibility of the radiomics features, one of the radiologists randomly selected a total of 30 cases for a second round of tumor segmentation, and the intra-class correlation coefficient (ICC) between the double annotations was calculated.

### Extraction and dimensionality reduction of radiomics features

Before extracting radiomics features, CT images underwent resampling to a uniform 1.0 mm*1.0 mm*1.0 mm resolution. Additionally, CT images were discretized with a binwidth set to 25. These procedures were implemented to ensure the reproducibility of radiomics features. Radiomics features were derived from corticomedullary phase CT images using the open-source software FeAture Explorer (version 0.5.5) [17]. These features were categorized into three distinct groups: (1) The first group, referred to as first-order features, characterizes the distribution of voxel intensity within CT images. This group provides insights into voxel symmetry, uniformity, and local intensity distribution variations; (2) The second group comprises morphological features, which capture information about the shape and size of the tumor lesion; (3) The third group includes texture features, comprising the gray-level co-occurrence matrix (GLCM), gray-level run-length matrix (GLRLM), gray-level size zone matrix (GLSZM), neighborhood gray-tone difference matrix (NGTDM), and gray-level dependence matrix (GLDM). In total, 107 radiomics features were extracted for each lesion, including 14 shape features, 18 first-order features, and 75 texture features.

**Fig. 3:**
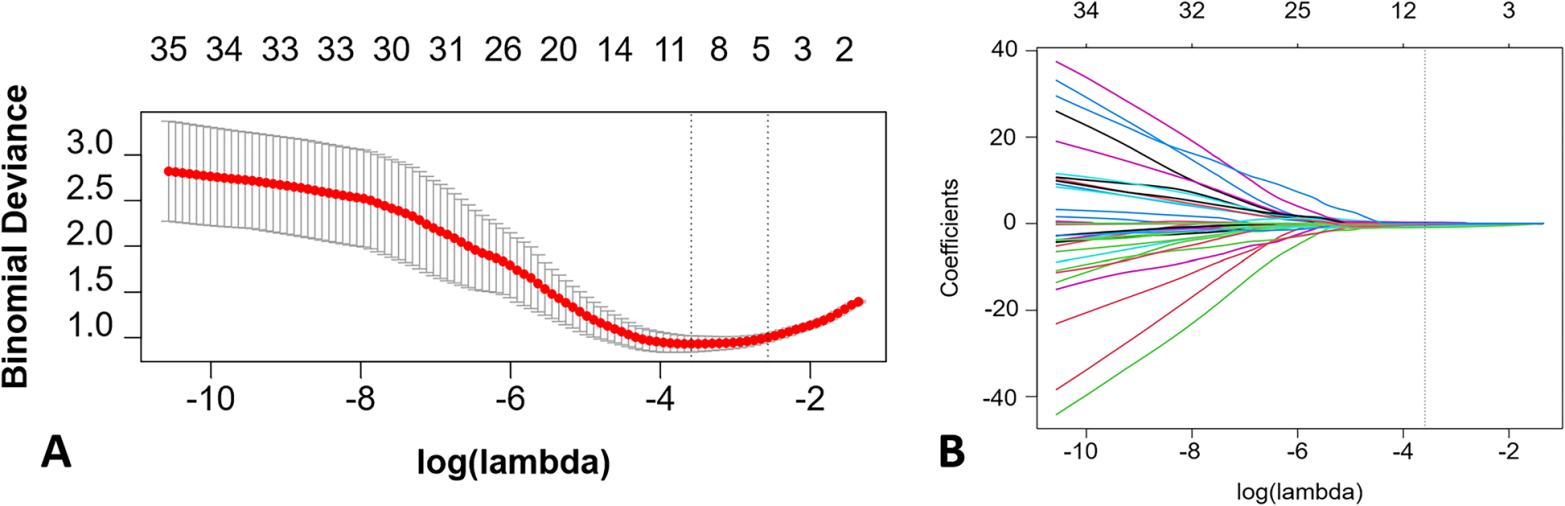
LASSO screening pathway. Figure **A** shows the selection of the optimal hyperparamter λ with the minimal binomial deviance, identified through a five-fold cross-validation process; Figure **B** illustrates the selection of radiomics features under the optimal hyperparamter λ

However, some radiomics features may exhibit minimal distinctiveness and contribute little to the final outcome. Hence, a necessary step involved downscaling and retaining the most relevant radiomics features in the training set. All radiomics features underwent normalization using the z-score normalization method. Furthermore, radiomics features with an ICC greater than 0.80 between double annotations were selectively retained to ensure the robustness of the radiomics features. Subsequently, to address imbalanced class distribution in the training set, the Synthetic Minority Over-Sampling Technique (SMOTE) was employed. To mitigate correlations among radiomics features, Pearson correlation coefficient (PCC) values were calculated for pairwise radiomics features. Any radiomics features with a PCC value exceeding 0.90 were randomly eliminated. Then, the Least Absolute Shrinkage and Selection Operator (LASSO) method was employed for feature selection. The optimal hyperparameter λ, identified through a five-fold cross-validation process, was selected based on the minimal predictive error (Fig. 3). Finally, a multivariate stepwise logistic regression was used for selecting the final optimal radiomics features with *P*-values less than 0.05, and the odds ratio (OR) and 95% confidence interval (CI) of the selected radiomics features were calculated.

### Model building

Utilizing radiomics features selected through multivariate stepwise logistic regression along with their corresponding coefficients, we calculated the radiomics score (Radscore) and subsequently constructed the radiomics model employing a logistic regression algorithm utilizing the open-source software FeAture Explorer (version 0.5.5). The tol, c, and max_iter parameters of the logistic regression algorithm were 0.01, 1.0, and 100, respectively. To assess the model's performance, we conducted cross-validation within the training set using a fivefold cross-validation approach, followed by validation in the test set. We employed metrics, including the receiver operating characteristic (ROC) curve, area under the curve (AUC), 95% CI, accuracy, sensitivity, specificity, and F1 score as key indicators for evaluating the model's effectiveness. Moreover, we employed calibration curve to evaluate the model's goodness of fit and utilized clinical decision curve and clinical impact curve to assess the model's clinical value.

### Image analysis

**Fig. 4:**
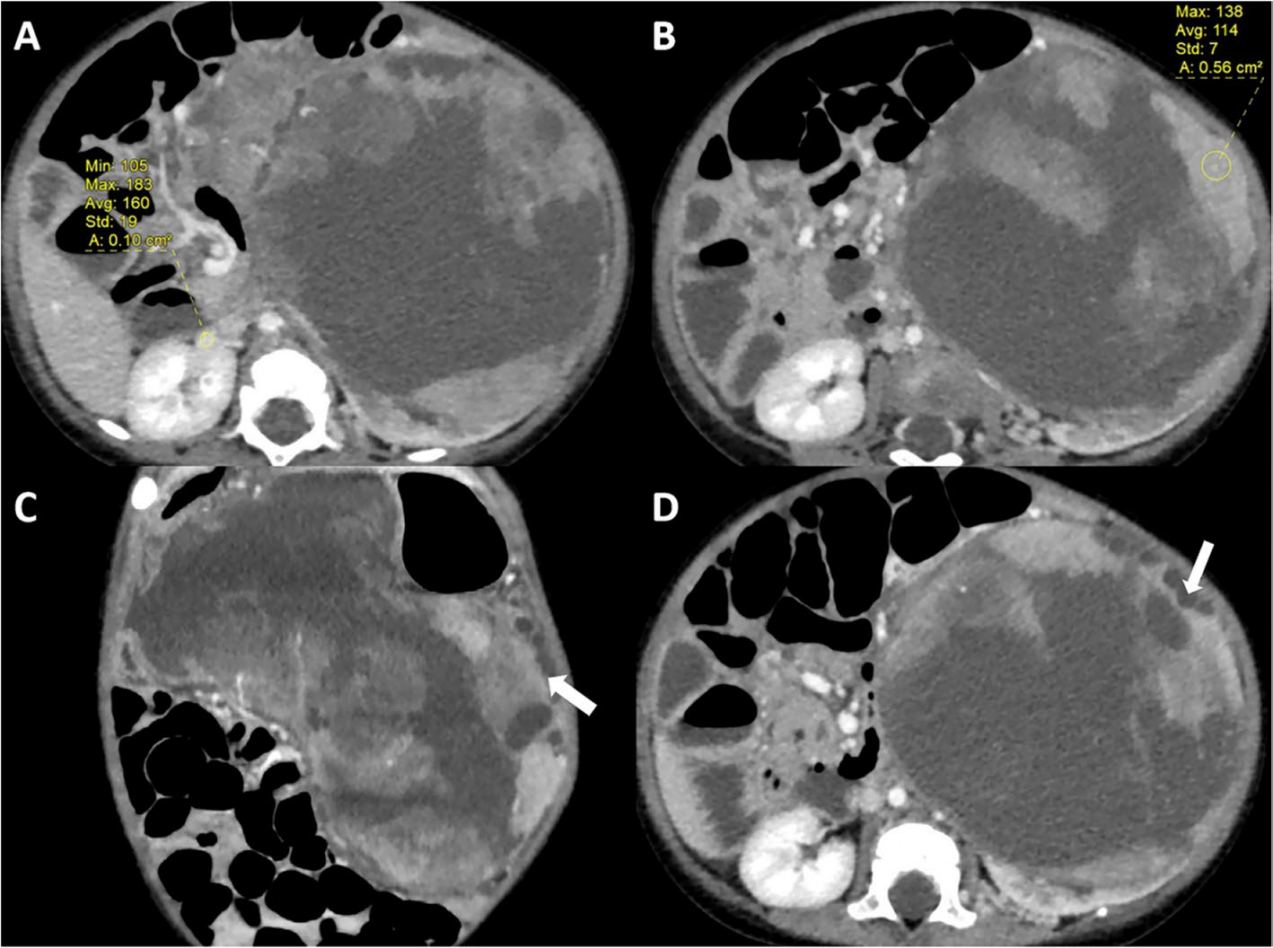
Examples of image analysis. Figure **A** shows the placement of a circular region of interest on the renal vein contralateral to the mass for measuring CT values; Figure **B** shows the placement of a circular region of interest on the most obviously enhanced part of the tumor parenchyma for measuring CT values; Figures **C** and **D** show the presence of dilated peritumoral cysts (white arrows)

An experienced pediatric radiologist evaluated several imaging features from the nephrographic phase, including the maximum tumor diameter, the ratio of the maximum CT value of the tumor solid portion to the mean CT value of the contralateral renal vein (CTmax/CT renal vein), and the presence of dilated peritumoral cysts. A previous study revealed that CTmax/CT renal vein was particularly valuable in distinguishing between CCSK and WT in children under 10 years of age using contrast-enhanced CT imaging [7]. In this context, CTmax referred to the maximum CT value within the most conspicuously enhancing region during the nephrographic phase of the tumor. Meanwhile, CT renal vein was defined as the mean CT value of the contralateral renal vein. To mitigate measurement error, each parameter was measured three times, with the mean value subsequently recorded (Fig. 4A and B). Dilated peritumoral cysts were defined as areas within the tumor that exhibited slight hypointensity in the un-enhanced phase, followed by the appearance of at least three such areas in different directions during the enhancement phase, which remained unenhanced at the tumor margins (Fig. 4C and D).

### Statistical analysis

Statistical analysis was conducted using SPSS software (version 26.0) and RStudio software (version 4.2.2). Continuous variables underwent Shapiro–Wilk test and Levene's test to verify their adherence to normal distribution and homogeneity of variance, respectively. When these prerequisites were satisfied, the analysis proceeded using Student’s t-test. However, if either the assumption of normality or homogeneity of variance was violated, the non-parametric Mann–Whitney U test was employed as an alternative. For categorical variables, the chi-square test was utilized when applicable, and in instances where the expected counts were small, the Fisher's exact test was employed. To assess the stability of the radiomics model against various assumptions and methodological choices, we conducted a sensitivity analysis using a stepwise elimination approach on the employed feature selection methods. A two-tailed *P*-value of less than 0.05 was considered indicative of statistically significant differences.

## Results

### Basic clinical information

In this study, a total of 116 patients were enrolled, comprising 83 WT cases and 33 CCSK cases. These patients were further stratified into a training set, consisting of 81 cases (58 WT and 23 CCSK), and a test set, comprising 35 cases (25 WT and 10 CCSK), following a 7:3 ratio allocation. Table 1 presents the basic clinical information for WT and CCSK patients in both the training and test sets. Among these patients, 63 (54.3%) were males, while 53 (45.7%) were females, with a mean and standard deviation age of 2.58 years and 2.10 years, respectively, spanning from 2 months to 9 years. Both age and gender exhibited no statistically significant differences between the groups in the training and test sets (*P* > 0.05).

**Table 1:** Comparison of basic clinical information and imaging characteristics of WT and CCSK in the training and test sets

**Characteristics**	**Training set (*n* = 81)**			**Test set (*n* = 35)**		
	**WT (*n* = 58)**	**CCSK (*n* = 23)**	***P* value**	**WT (*n* = 25)**	**CCSK (*n* = 10)**	***P* value**
Gender			0.270			0.060
Male	30	15		10	8	
Female	28	8		15	2	
Age (years, mean $\pm$ std)	2.48 $\pm$ 1.94	2.27 $\pm$ 2.18	0.297	3.05 $\pm$ 2.20	2.80 $\pm$ 2.65	0.465
Maximum tumor diameter (mm, mean $\pm$ std)	86.19 $\pm$ 30.82	103.43 $\pm$ 26.24	0.021	94.54 $\pm$ 21.13	88.09 $\pm$ 32.06	0.488
Presence of dilated peritumoral cyst			0.005			0.107
Yes	14	13		5	5	
No	44	10		20	5	
CTmax/CT renal vein	0.77 $\pm$ 0.21	0.86 $\pm$ 0.24	0.091	0.68 $\pm$ 0.16	0.77 $\pm$ 0.27	0.217

*WT* Wilms tumor, *CCSK* Clear cell sarcoma of the kidney; std, standard deviation

### CT imaging characteristics

In the training set, there were statistically significant differences in the maximum tumor diameter (*P* = 0.021) and the presence of dilated peritumoral cysts (*P* = 0.005) between WT and CCSK, whereas in the test set, no statistically significant differences were observed (*P* > 0.05). However, the CTmax/CT renal vein did not exhibit statistically significant differences between the groups in either the training set or the test set (*P* > 0.05) (Table 1).

### Radiomics feature extraction and dimensionality reduction

**Fig. 5:**
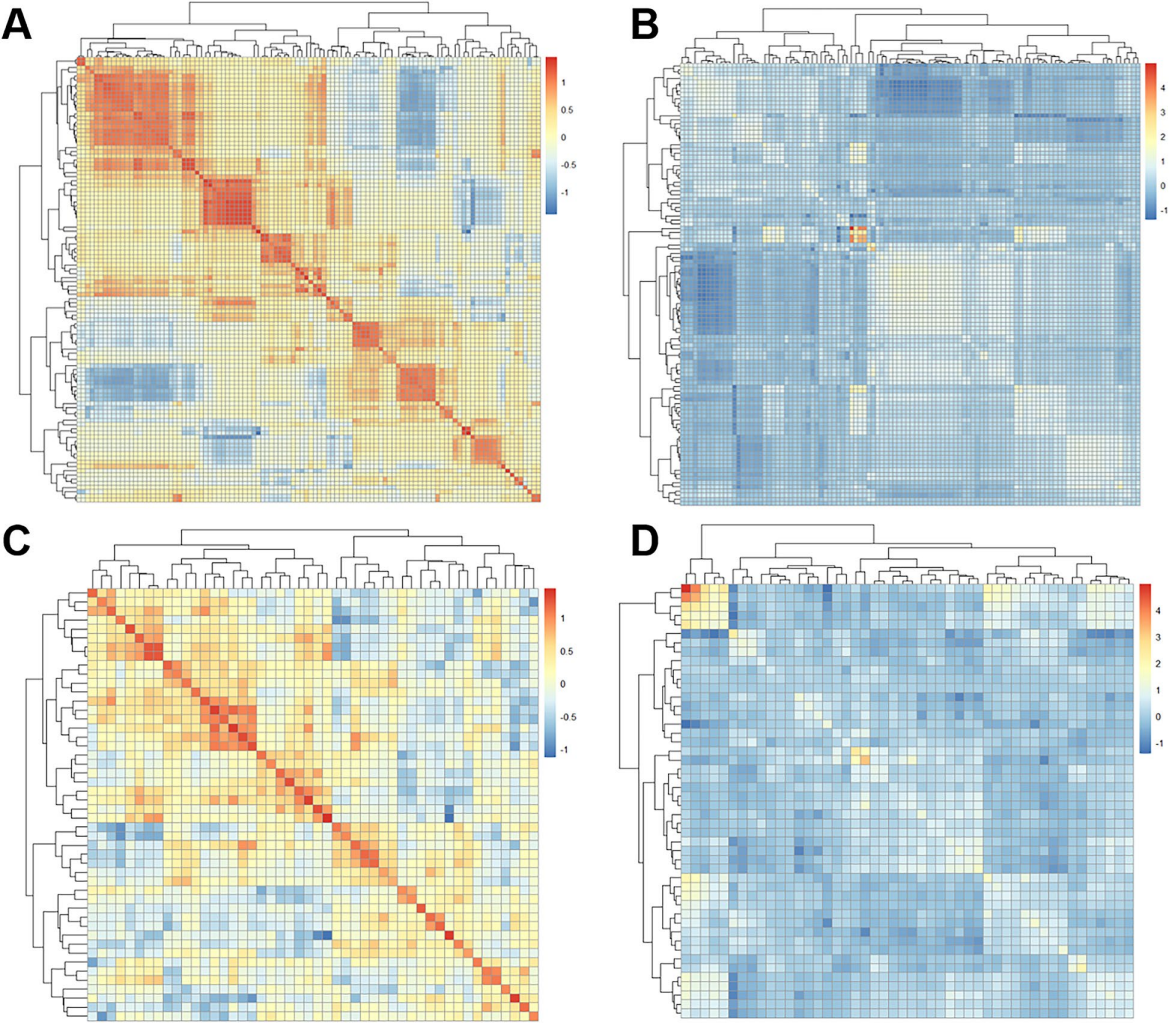
Heatmaps of radiomics features before and after Pearson correlation analysis. Figures **A** and **B** show heatmaps of radiomics features before Pearson correlation analysis in the training and test sets, respectively; Figures **C** and **D** show heatmaps of radiomics features after Pearson correlation analysis in the training and test sets, respectively

**Fig. 6:**
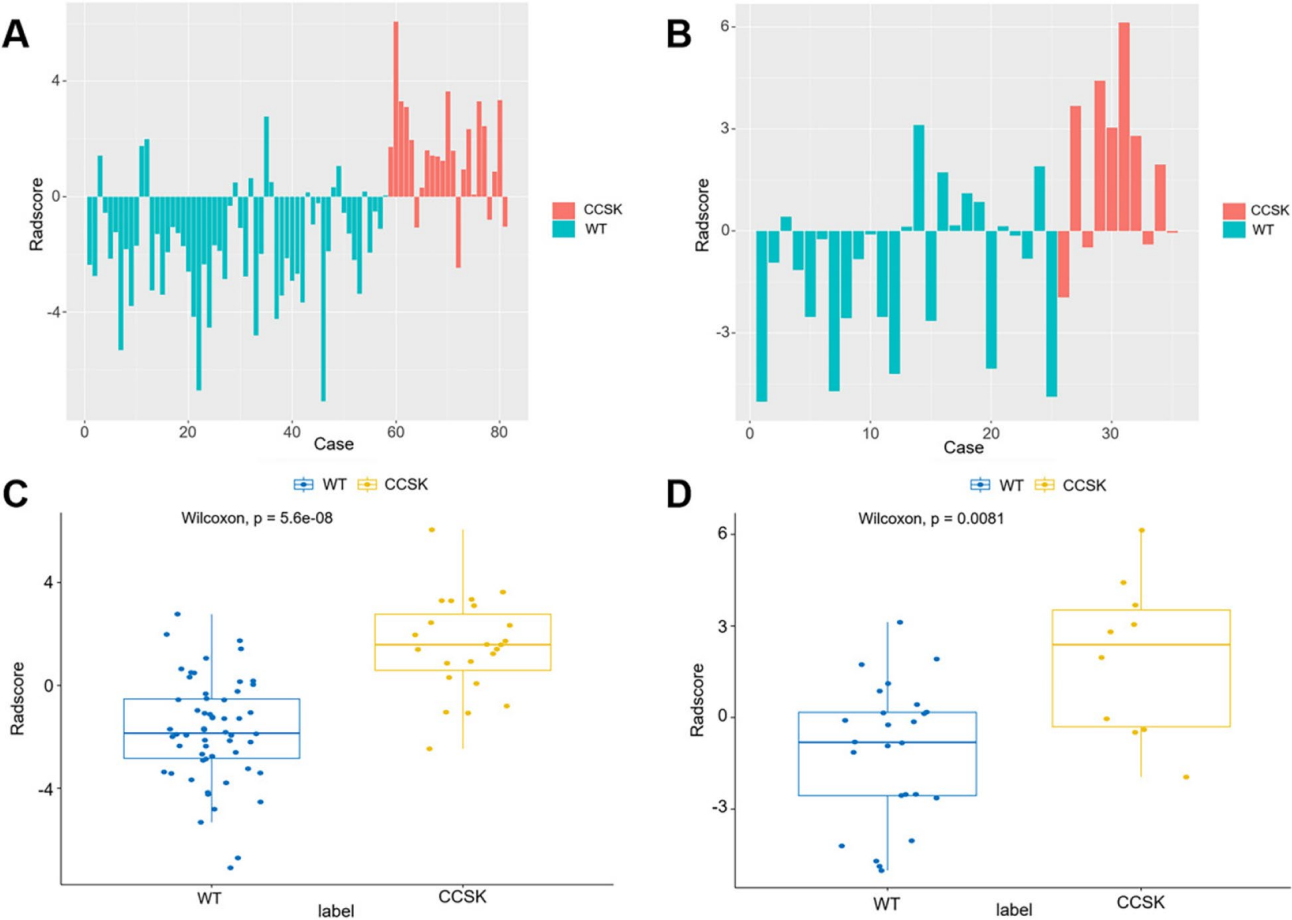
The distribution of Radscore. Figures **A** and **B** show the Radscore of each case in the training and test set, respectively; Figures **C** and **D** show the comparison of Radscore between groups in the training and test set, respectively

For each lesion, a total of 107 radiomics features were initially extracted. The average and standard deviation of the ICC values for all radiomics features between the double annotations were 0.95 and 0.06, respectively. Subsequently, one feature was eliminated using an ICC threshold of 0.80, and an additional 58 features were removed based on a PCC threshold of 0.90 (Fig. 5). Then, nine radiomics features were selected through LASSO pathway (Fig. 3). Finally, four optimal radiomics features with *P*-values less than 0.05 were retained (Table 2). The inter-correlation of the selected radiomics features are demonstrated in Supplementary Fig. 1. Based on the following formula, Radscore was computed for both the training and test sets. There was a statistical difference in Radscore between CCSK and WT in both the training and test sets (*P* < 0.05) (Fig. 6). The selected radiomics features and calculated Radscore did not differ significantly between training set and test set (*P* > 0.05) (Supplementary Fig. 2).

**Table 2:** The final selected radiomics features and their corresponding coefficients following the multivariate stepwise logistic regression

Categories	Subcategories	Coefficient	OR (95%CI)	*P* value
firstorder	Median	-0.751	0.472 (0.237–0.941)	0.033
glcm	MaximalCorrelationCoeffcient	-0.937	0.392 (0.216–0.713)	0.002
glszm	ZoneEntropy	-0.759	0.468 (0.247–0.887)	0.020
shape	SurfaceVolumeRatio	-1.462	0.232 (0.091–0.590)	0.002

*CI* Confidence interval, *glcm* Gray-level co-occurrence matrix, *glszm* Gray-level size zone matrix, *OR* Odds ratio

**Table:** 

Radscore = -0.076-A × 0.751-B × 0.937-C × 0.759-D × 1.462
A = firstorder_Median
B = glcm_MaximalCorrelationCoeffcient
C = glszm_ZoneEntropy
D = shape_SurfaceAreatoVolumeRatio

### Discriminative efficacy of radiomics model

**Fig. 7:**
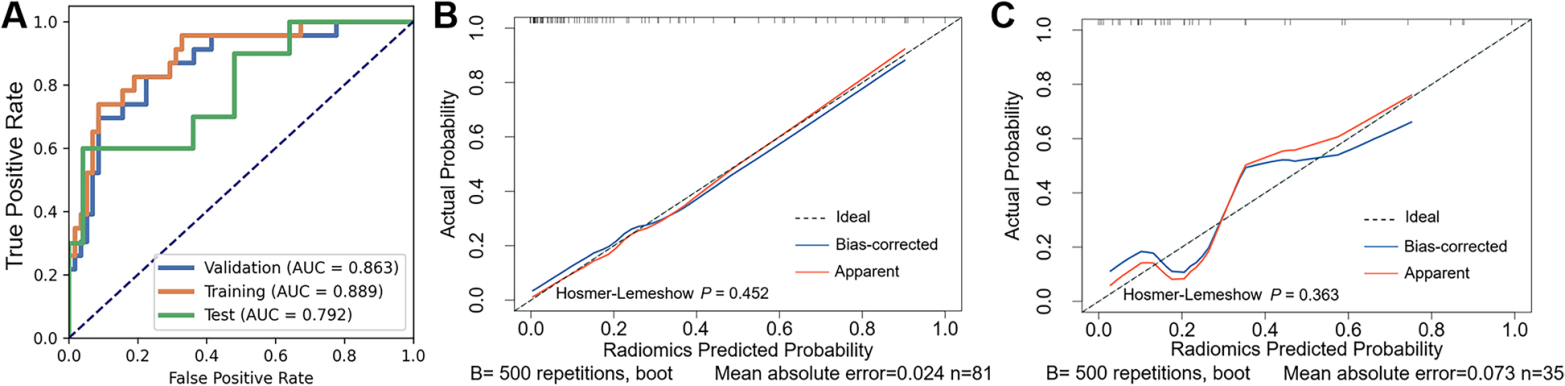
Diagnostic performance of the radiomics model. Figure **A** show the receiver operating characteristic curves of the radiomics model in the training and test set; Figures **B** and **C** illustrate the calibration curves of the radiomics model in the training and test set, respectively

**Fig. 8:**
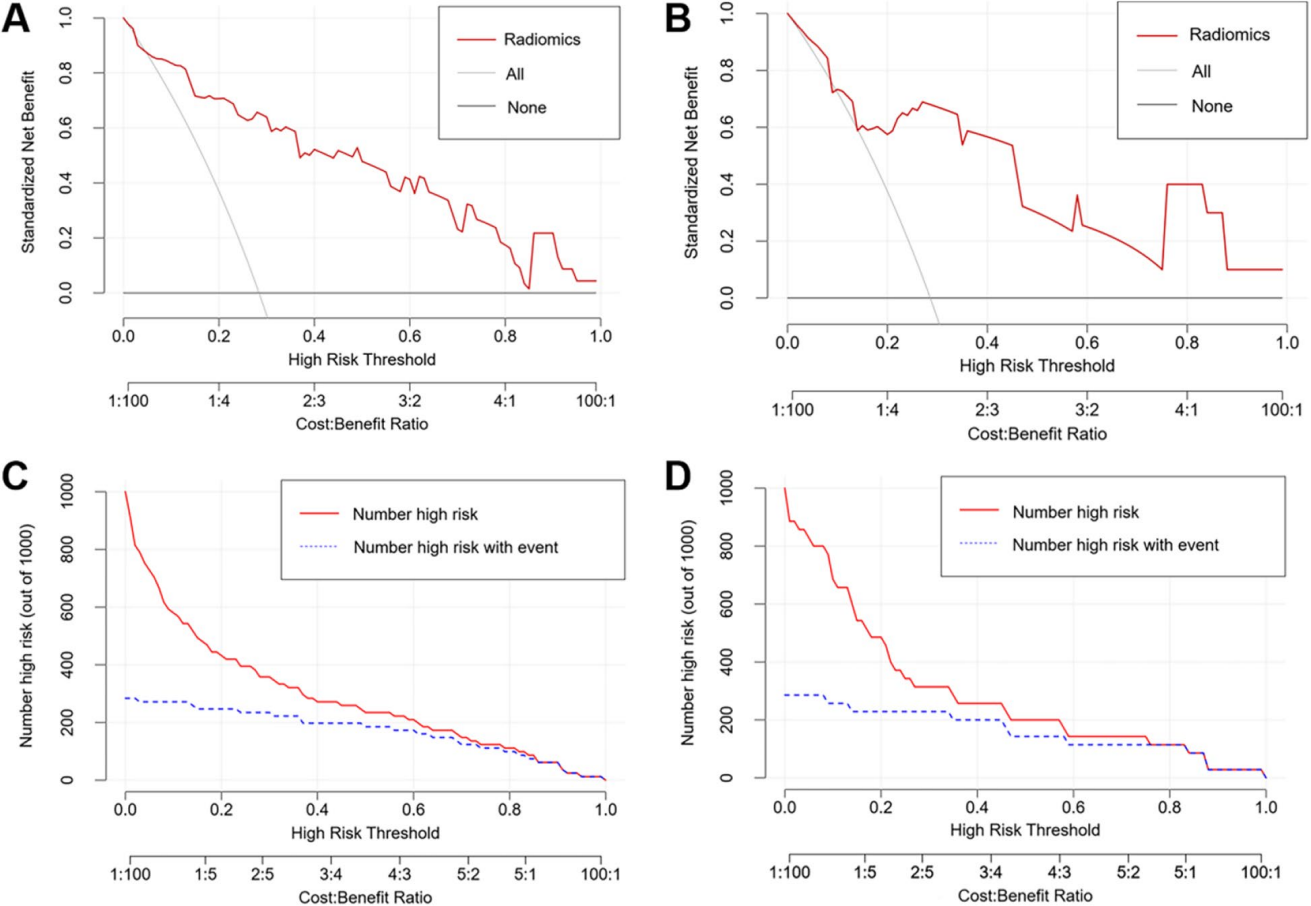
Clinical utility of the radiomics model. Figures **A** and **B** show the clinical decision curves of the radiomics model in the training and test set, respectively; Figures **C** and **D** illustrate the clinical impact curves of the radiomics model in the training and test set, respectively

The radiomics model exhibited strong performance in the training set, with an AUC of 0.889 (95% CI: 0.811–0.967), along with an accuracy of 0.864, a sensitivity of 0.739, and a specificity of 0.914. Furthermore, when assessed via fivefold cross-validation in the training set, the model consistently displayed a high AUC of 0.863 (95% CI: 0.774–0.952), along with an accuracy of 0.852, a sensitivity of 0.696, and a specificity of 0.914. In the test set, the radiomics model maintained favorable performance, achieving an AUC of 0.792 (95% CI: 0.616–0.968), an accuracy of 0.857, a sensitivity of 0.600, and a specificity of 0.960. Additional evaluation metrics can be found in Table 3. The ROC and calibration curves for the radiomics model in both the training and test sets are depicted in Fig. 7, indicating favorable diagnostic performance and goodness of fit. Additionally, Fig. 8 shows the clinical decision curves and clinical impact curves for the radiomics model in both sets, which demonstrated the clinical utility of the radiomics model. When the stepwise elimination approach was applied on the employed feature selection methods, there was no significant difference in the diagnostic performance of the radiomics models, indicating the robustness of the radiomics model against various feature selection methods (Table 4).

**Table 3:** Evaluation parameters of the radiomics model

Parameters	Training set	fivefold CV in the training set	Test set
AUC (95% CI)	0.889 (0.811–0.967)	0.863 (0.774–0.952)	0.792 (0.616–0.968)
Accuracy	0.864	0.852	0.857
MCC	0.662	0.627	0.634
Sensitivity	0.739	0.696	0.600
Specificity	0.914	0.914	0.960
PPV	0.773	0.762	0.857
NPV	0.898	0.883	0.857
Youden Index	0.653	0.609	0.560
F1 score	0.756	0.728	0.706

*AUC* Area under the curve, *CI* Confidence interval, *MCC* Matthews correlation coefficient, *PPV* Positive prediction value, *NPV* Negative prediction value, *CV* Cross-validation

**Table 4:** A comparison of radiomics model performance when the stepwise elimination approach was applied on the employed feature selection methods

Statistical method eliminated	Training set					Test set				
	**AUC**	**95% CI**	**Accuracy**	**Sensitivity**	**Specificity**	**AUC**	**95% CI**	**Accuracy**	**Sensitivity**	**Specificity**
ICC	0.889	0.811–0.967	0.864	0.739	0.914	0.792	0.616–0.968	0.857	0.600	0.960
PCC	0.889	0.811–0.967	0.864	0.739	0.914	0.792	0.616–0.968	0.857	0.600	0.960
LASSO	0.905	0.831–0.978	0.901	0.739	0.966	0.764	0.592–0.936	0.714	0.800	0.680
Multivariate stepwise logistic regression	0.901	0.826–0.976	0.889	0.826	0.914	0.784	0.604–0.964	0.829	0.600	0.920
Combined	0.889	0.811–0.967	0.864	0.739	0.914	0.792	0.616–0.968	0.857	0.600	0.960

*AUC* Area under the curve, *CI* Confidence interval, *ICC* Intraclass correlation coefficient, *PCC* Pearson correlation coefficient, *LASSO* Least Absolute Shrinkage and Selection Operator

## Discussion

In this study, we investigated the effectiveness of radiomics features extracted from corticomedullary phase CT images in distinguishing between CCSK and WT in pediatric patients. Initially, we conducted an analysis of the qualitative and semi-quantitative imaging characteristics of CCSK and WT. Our findings revealed that, within the training set, there were statistically significant differences in the maximum tumor diameter and the presence of dilated peritumoral cysts between WT and CCSK. However, no statistically significant differences were observed in the test set, and this may be caused by small sample size in the test set. Nonetheless, Radscore exhibited significant differences between CCSK and WT in both the training and test sets. This underscores the added value of radiomics features in the differentiation of CCSK and WT. Furthermore, the constructed radiomics model demonstrated favorable diagnostic performance, a good fit, and clinical utility.

In conventional imaging, CCSK can appear quite similar to WT. WT typically manifests as large masses, often exhibiting necrosis and hemorrhage within the tumor. These tumors tend to have less intense imaging characteristics [18]. In contrast, CCSK often presents with more congested vessels and intensified tumors, sometimes displaying "tiger spot" changes [7]. Additionally, CCSK has a propensity for extra-renal metastases, particularly in the bones and brain. In this study, while the maximum tumor diameter and CTmax/CT renal vein did not show statistically significant differences, the presence of the dilated peritumoral cysts exhibited statistical significance in the training set. This finding may be attributed to factors such as dilated tubules or changes in the tumor capsule [19]. However, due to the limited sample size in the test set, there were no statistically significant differences in these imaging findings between CCSK and WT. Further studies with larger sample sizes are still needed to validate the diagnostic potential of these imaging findings.

However, our study revealed significant differences in the Radscore between CCSK and WT in both the training and test sets. This suggests that quantitative radiomics features derived from contrast-enhanced CT images are more adept at capturing the imaging differences between CCSK and WT compared to visual analysis, and they play a pivotal role in the differential diagnosis of these two diseases. Traditional qualitative or semi-quantitative imaging features often fall short in capturing the full extent of heterogeneity within the tumor. In recent years, radiomics has emerged as a promising approach for quantitatively evaluating tumor phenotypes [20, 21]. In our study, we employed a CT radiomics approach for the first time to differentiate between CCSK and WT. In the clinical practice, accurate identification of CCSK and WT can potentially lead to the avoidance of unnecessary 4-week administration of Vincristin/Actinomycin D in CCSK patients, thereby enhancing the clinical management of these patients.

In previous studies, first-order histogram features of renal tumors have exhibited variations among different pathological types [15, 22, 23]. For instance, Deng et al. [23] investigated the role of CT texture analysis in distinguishing the pathological staging of major renal cell carcinomas. They found that first-order entropy could help differentiate clear cell from papillary renal tumors, and skewness and kurtosis were also identified as valuable in distinguishing clear cell renal carcinomas from eosinophilic tumors. However, in other studies focusing on texture analysis of non-renal tumors, second-order features, such as GLSZM or GLDM, appear to play a significant role in characterizing the heterogeneity of non-renal tumors [14, 24, 25]. These studies have demonstrated that certain GLDM texture metrics of intratumor and peritumor fat can quantitatively and distinctly distinguish between urothelial carcinoma and micropapillary carcinoma [24]. In our study, the final selection of radiomics features predominantly comprised texture features. This emphasizes the diagnostic potential of inhomogeneity and heterogeneity within image voxels. The chosen texture features mainly reflect attributes related to geometric patterns, the presence of image gray values, and the coexistence of image gray values, respectively.

Prior studies have indicated that the choice of feature selection methods can have varying impacts on the efficacy of constructing models in radiomics research [26, 27]. In our investigation, we opted for PCC, LASSO, and stepwise logistic regression to select radiomics features in order to enhance the model's generalizability. Previous investigation has proposed that employing simpler statistical techniques for radiomics feature selection may enhance the model's robustness and reproducibility [28]. Furthermore, the performance of the radiomics model can also fluctuate depending on the choice of machine learning methods [25]. In our preliminary experiments, we explored various machine learning algorithms, including support vector machine and random forest, but ultimately, the model constructed using the logistic regression algorithm exhibited superior performance.

From a therapeutic perspective, this study offers several clinical advantages. Firstly, it helps in avoiding preoperative biopsies that can potentially disseminate tumor cells [29]. Secondly, it mitigates diagnostic bias in needle biopsies stemming from the spatial heterogeneity within tumors [30]. Lastly, Wilde et al. [31] demonstrated that stage I WT had the potential for renal preservation since tumor cells did not invade the renal sinus and blood vessels, allowing for unit-preserving nephrectomy. Similarly, stage I CCSK could benefit from less intensive chemotherapy [32], offering more clinical treatment options. In our investigation, the radiomics model exhibited superior diagnostic efficacy in distinguishing CCSK from WT. This was validated not only through calibration curves but also clinical decision curves and clinical impact curves.

This study has several limitations. Firstly, it is important to acknowledge that both CCSK and WT are clinically rare, with CCSK being even rarer than WT. Over an 11-year period, this study included a relatively small sample size of 83 children with WT and 33 children with CCSK, which may raise concerns about the statistical power and generalizability of the findings. Nevertheless, our study underscores the clinical significance of CT radiomics in distinguishing between CCSK and WT. Secondly, the use of SMOTE, like any data augmentation method, can introduce certain biases and potentially inflate model performance. To address this concern, it's crucial to assess the model's performance on independent datasets to gauge the potential biases introduced by SMOTE. In this study, we employed five-fold cross-validation in the training set and further validated the model's performance in the test set. The results highlighted the model's favorable diagnostic performance. Lastly, given the relative rarity of WT and CCSK and the associated challenges in case collection, this study did not incorporate external validation. As a result, the model's generalization capability remains to be confirmed, and the next step involves the collection of multi-center data for external validation purposes.

In summary, quantitative radiomics features derived from corticomedullary phase CT images are instrumental in the differentiation between CCSK and WT. They outperform qualitative or semi-quantitative imaging features obtained through visual analysis. The radiomics model can aid in the differentiation of malignant tumors in pediatric patients. It yields clinical advantages under various risk thresholds, potentially offering additional diagnostic biomarkers for pediatric malignant renal tumors.

### Supplementary Information

**Additional file 1.**

## Data Availability

The datasets generated or analyzed during the current study are not publicly available, but are available from the corresponding author on reasonable request.
