# Supplementary material for: CT radiomics to differentiate between Wilms tumor and clear cell sarcoma of the kidney in children
Source: BMC Med Imaging. 2024 Jan 5;24:13. doi: 10.1186/s12880-023-01184-2 (PMC10768092; doi:10.1186/s12880-023-01184-2)
Supplement: Supplementary file 1 — Additional file 1. [file 12880_2023_1184_MOESM1_ESM.docx]

**Supplementary Materials**


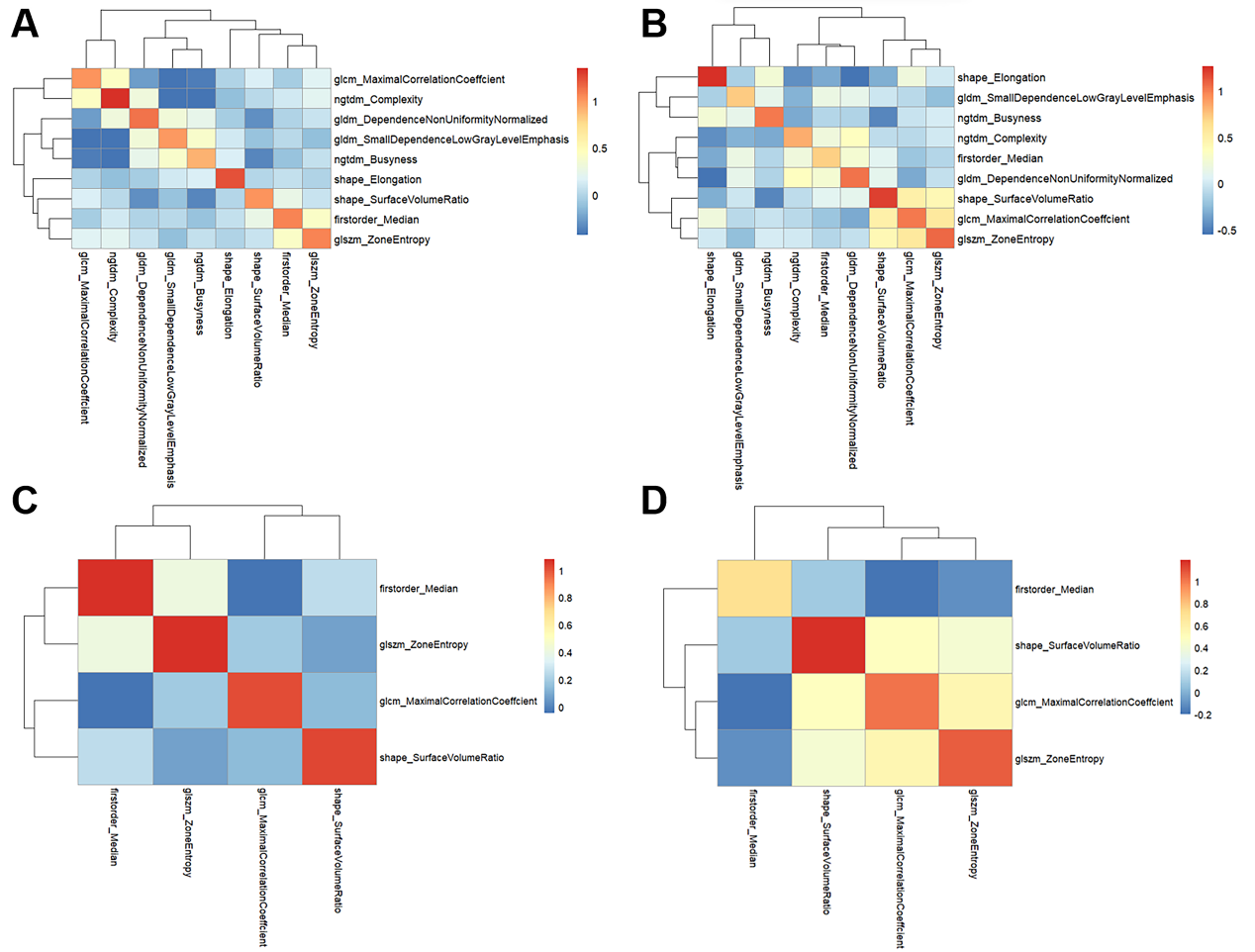


**Supplementary Figure 1** Inter-correlation of the radiomics features. Figures A and B show the heatmaps of radiomics features selected by least absolute shrinkage and selection operator in the training set and test set, respectively; Figures C and D show the heatmaps of radiomics features selected by multivariate stepwise logistic regression in the training set and test set, respectively.


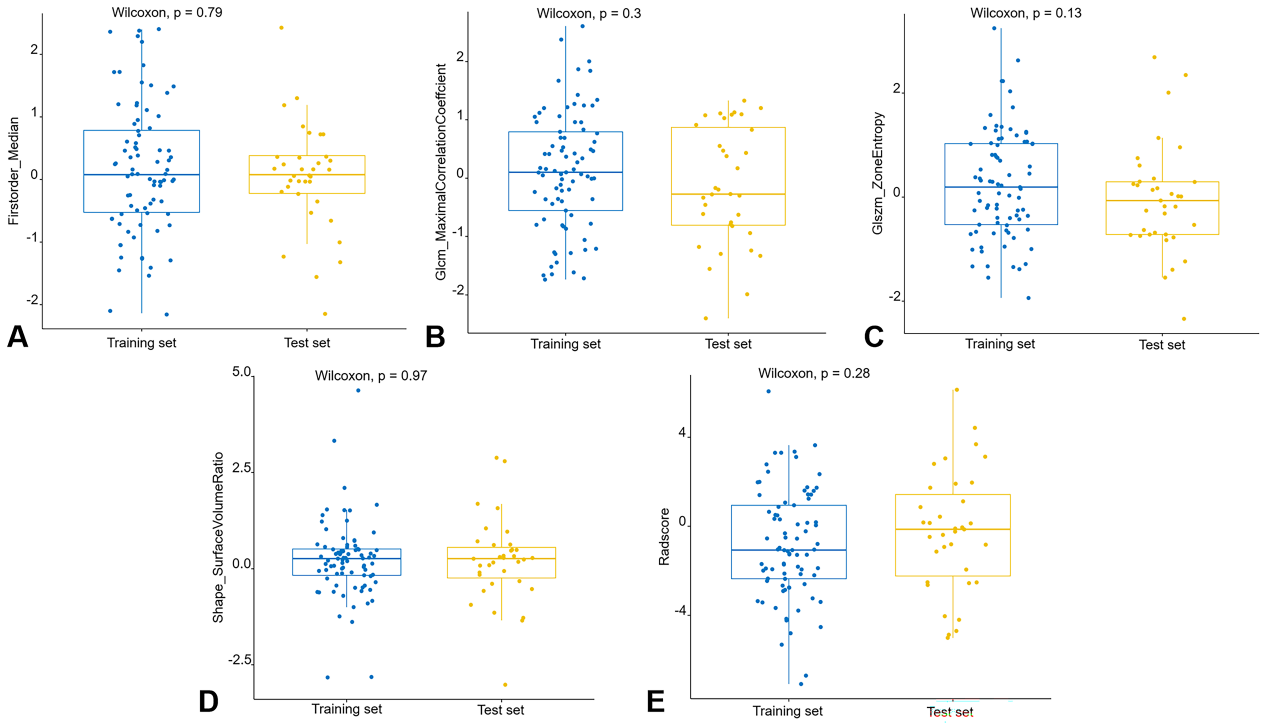


**Supplementary Figure 2** Comparisons of the final selected radiomics features and calculated Radscore between training set and test set.
